# A hybrid simulation model approach to examine bacterial genome sequencing during a hospital outbreak

**DOI:** 10.1186/s12879-019-4743-3

**Published:** 2020-01-23

**Authors:** Thomas M. Elliott, Xing J. Lee, Anna Foeglein, Patrick N. Harris, Louisa G. Gordon

**Affiliations:** 1 Population Health Department, QIMR Berghofer Medical Research Institute, 300 Herston Rd, Herston, Brisbane, Q4006 Australia; 2 Australian Centre for Health Services Innovation, School of Public Health and Social Work, Queensland University of Technology, Kelvin Grove, Brisbane, 4059 Australia; 3 Heisenberg Analytics, Indooroopilly, QLD 4068 Australia; 4 Central Microbiology, Pathology Queensland, Royal Brisbane and Women’s Hospital, Herston, QLD Australia; 5 Faculty of Medicine, UQ Centre for Clinical Research, The University of Queensland, Herston, QLD Australia; 6 School of Medicine, The University of Queensland, Brisbane, Australia; 7 School of Nursing, Queensland University of Technology, Kelvin Grove, Brisbane, Q4059 Australia

**Keywords:** Simulation modeling, Agent-based model, Discrete-event model, Pathogen sequencing, Whole genome sequencing, Hospital outbreak

## Abstract

**Background:**

Hospital infection control requires timely detection and identification of organisms, and their antimicrobial susceptibility. We describe a hybrid modeling approach to evaluate whole genome sequencing of pathogens for improving clinical decisions during a 2017 hospital outbreak of OXA-181 carbapenemase-producing *Escherichia coli* and the associated economic effects.

**Methods:**

Combining agent-based and discrete-event paradigms, we built a hybrid simulation model to assess hospital ward dynamics, pathogen transmission and colonizations. The model was calibrated to exactly replicate the real-life outcomes of the outbreak at the ward-level. Seven scenarios were assessed including genome sequencing (early or late) and no sequencing (usual care). Model inputs included extent of microbiology and sequencing tests, patient-level data on length of stay, hospital ward movement, cost data and local clinical knowledge. The main outcomes were outbreak size and hospital costs. Model validation and sensitivity analyses were performed to address uncertainty around data inputs and calibration.

**Results:**

An estimated 197 patients were colonized during the outbreak with 75 patients detected. The total outbreak cost was US$318,654 with 6.1% of total costs spent on sequencing. Without sequencing, the outbreak was estimated to result in 352 colonized patients costing US$531,109. Microbiology tests were the largest cost component across all scenarios.

**Conclusion:**

A hybrid simulation approach using the advantages of both agent-based and discrete-event modeling successfully replicated a real-life bacterial hospital outbreak as a foundation for evaluating clinical outcomes and efficiency of outbreak management. Whole genome sequencing of a potentially serious pathogen appears effective in containing an outbreak and minimizing hospital costs.

## Introduction

Nosocomial infections, also known as healthcare-associated infections (HAI), affect 7.1 patients per 100 patients (95% confidence interval: 6.5–7.8) in high-income countries [1]. The increasing presence of multidrug resistant pathogens is compounding the importance of reducing nosocomial infections. HAI confirmation currently relies on the results of microbiology cultures and, less frequently, molecular assays. The lack of specificity in these tests causes inefficient or incorrect use of the limited isolation rooms available in major hospitals, particularly during HAI outbreaks. Bacterial whole genome sequencing (WGS) provides infection control teams with more granular information to better discern between different strains of bacteria which, in turn, can improve the way patients are managed during outbreaks, for example, better prioritizing patients for isolation [2]. Increase in world travel is also increasing the diversity of bacterial strains present in any one location. This may increase the risk of high-risk pathogens or novel antibiotic resistance genes becoming established in the population before being recognized.

Hospitals are continually under pressure to provide efficient and effective services with limited resources. Information on the direct costs, opportunity costs and patient health benefits for alternative courses of action assists decision-makers to make well-informed choices. Ineffective decisions could introduce higher costs and reduce opportunities to prevent morbidity or save lives [3]. Simulation modeling has a rich history in answering ‘what-if’ questions in complex environments such as hospitals where prospective trials or cohort studies are difficult, unethical or too expensive. Designing the appropriate simulation model is integral to predicting the correct outcomes and making use of available data [4]. The model choice depends on the level of detail needed, the level of agent interaction and time scale of the intervention.

The merits of agent-based simulation (ABS) and discrete-event simulation (DES) are often debated in modeling how to manage non-communicable diseases [4]. A systematic review on ABS models found seven studies involving interventions for controlling HAIs, 14 on methicillin-resistant *Staphylococcus aureus* transmission and three on *Clostridium difficile* [5]. Simulation studies within hospitals typically employ DES models [6]. Hybrid ABS/DES models have been sparsely used in hospital settings or outbreak scenarios. Hybrid simulation is a growing field in healthcare and AnyLogic® is the only program which can integrate the ABS, DES and system dynamics paradigms [7].

The purpose of this study was to build a hybrid model that simulated hospital processes involved in controlling an infectious disease outbreak. The model was motivated by an evaluation of the impact of WGS in managing an outbreak of a novel *Escherichia coli* strain (OXA-181). We compared WGS as it was used in practice (delayed use) with earlier WGS, or not at all, to determine the relative costs and benefits of these strategies.

## Methods

### Purpose

The hybrid ABS/DES model was developed using AnyLogic® version 8.3.3 (XJ Technologies, St Petersburg, Russia). The model was designed to evaluate the effect of WGS in providing information to infectious disease physicians to better manage patients involved in a hospital outbreak. We replicated a real outbreak at a major hospital in south east Queensland, Australia and simulated the effects of alternative WGS scenarios in controlling the outbreak. The model description hereunder follows the ‘Overview, Design concepts, and Details’ reporting guideline designed to facilitate a complete, rigorous and transparent agent-based model description [8].

#### The outbreak

The OXA-181 *E. coli* outbreak started in April of 2017 when a patient arriving from overseas was admitted to hospital. This patient was screened using usual microbiological tests, and was assumed to be colonized with a low-risk pathogen that did not require additional infection control precautions. The patient was permitted to move through the hospital unknowingly spreading the pathogen to other patients, until five patients were identified with the same species and resistance profile. Colonized patients were detected through the hospital’s routine infectious disease screening protocols. At this stage, WGS was performed on these five isolates. WGS identified the pathogen as sequence type (ST) 38 *Escherichia coli*, with close phylogenetic relatedness to strains previously identified in Vietnam. This strain also carried a gene encoding OXA-181 carbapenemase, which are rarely encountered in Australia and had not been previously described in Queensland public hospitals. They have rapidly emerged in some parts of the world, especially Turkey, North Africa and the Middle East [9, 10] and have been implicated in major hospital outbreaks [11].

### Entities, state variables, and scales

**Fig. 1:**
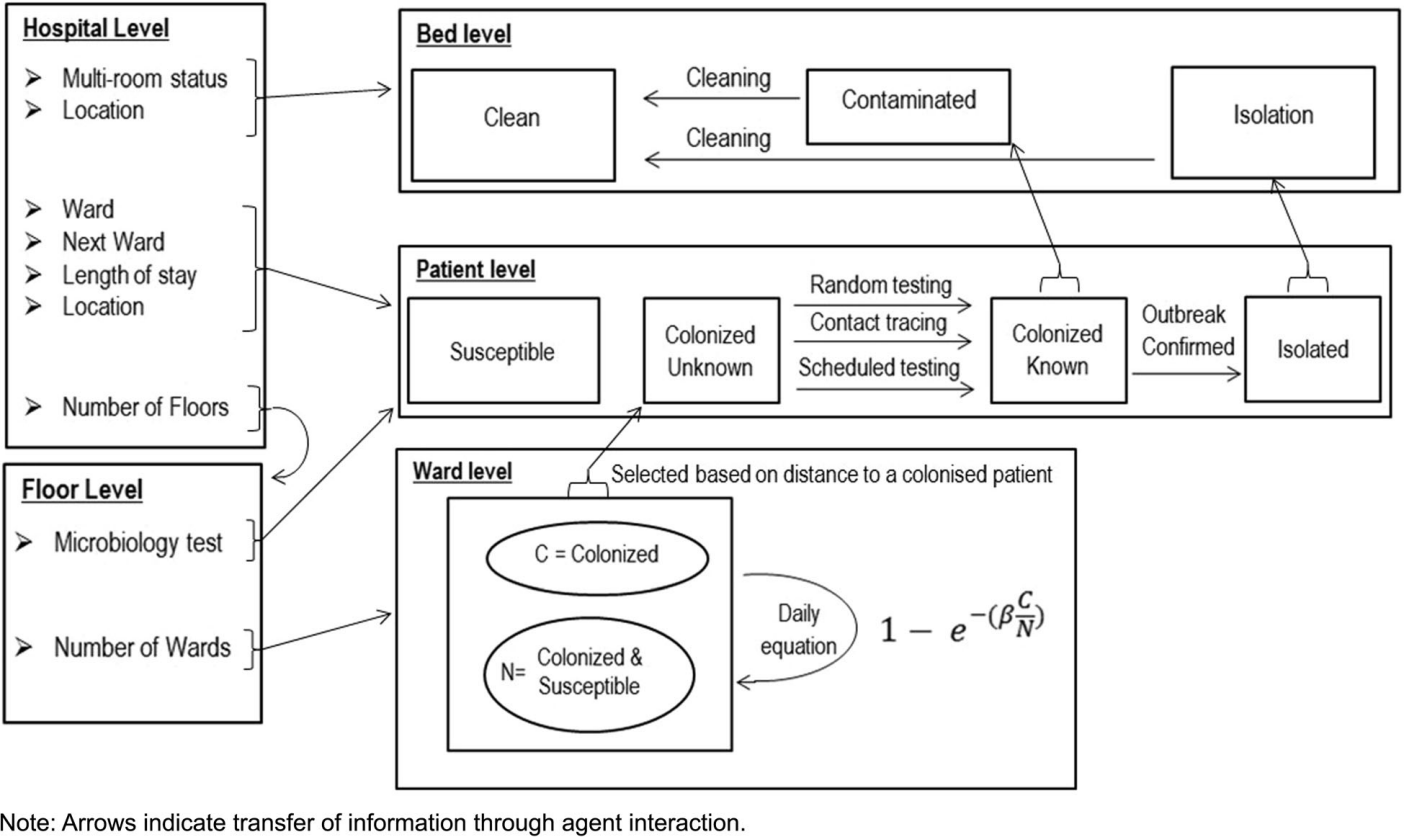
Schematic of hybrid simulation model and information pathway used in this evaluation

The hybrid model was constructed with mobile agents which represent the Patients, and static agents which represent the Beds, Wards and Hospital Floors. All four agent types exist within the hospital environment that controls the time, patient entry and spatial position (Fig. 1). The hierarchical approach was used when designing the hybrid model. Patient agents, Bed agents and each Floor agent exist within the main hospital environment which is the top level agent. Each ward exists within the Floor agents and Patients traversed the levels as they passed through the model. The patient flows, screening and bed management mechanisms were implemented using a DES approach. In contrast, the spread of the infection between wards was modelled with a state-chart based technique.

The Patient agent type represents the individuals being treated within the hospital. Each Patient has the following state variables:

1. Colonization status: susceptible, undetected colonized, detected colonized and infected. ‘Colonized’ means the person is asymptomatic but is harboring the pathogen while ‘infected’ means the person has the pathogen and is symptomatic.
2. Microbiology test: whether a test is required.
3. Room patient count: number of Patients in the same room.
4. Ward: current ward.
5. Next Ward: The ward the patient will be transferred to or lack of a ward, indicating discharge.
6. Length of stay: days to be spent in current ward.
7. Location: the geographic information system location of the Patient within the hospital environment.
8. Distance to closest colonized patient: If a colonized Patient is in the same Ward, the Patient will record the distance from that colonized Patient.

Each Bed holds certain information which affects how the Bed is managed during an outbreak:

1. Bed status: available for isolation, currently contaminated or already reserved for isolation.
2. Multi-room status: single bed or multi-bed room.
3. Location: the geographic information system location of the Bed within the hospital environment.

The role of each Ward agent is to calculate the spread of the pathogen. A Ward can either harbor the pathogen, where the spread is calculated daily or not. The Floor agent contains the Wards as per hospital design and controls the processes required to move Patients throughout the hospital.

### Process overview and scheduling

Processes within a hospital can take hours while others last days. A time unit of 1 hour was chosen based on the shortest frequency of events in the model. The model starts at day zero and ends when no new colonizations have been detected for 5 weeks. The model can be viewed on the AnyLogic™ cloud at https://cloud.anylogic.com/model/6fe44e5b-6276-44fd-95c8-ba93b3975262?mode=SETTINGS [12].

The hybrid model had three interacting sub-models; Hospital Mechanics, Outbreak Management and Pathogen Transmission. The three sub-models are described in detail in section 2.7. The Hospital Mechanics sub-model controls the patient journey through the use of DES. A patient enters the hospital, moves to their assigned ward, and stays there for a predetermined length of stay before being moved to another ward (if necessary) or discharged. This simple process is complicated by outbreak control measures which start once an outbreak is confirmed. The Outbreak Management sub-model may redirect patients from this simple process to facilitate infection control procedures such as screening or isolation. The Pathogen Transmission sub-model simulates the spread of the bacterial pathogen.

### Design concepts

The three main methods of simulation modeling are DES, ABS and system dynamics. Each technique has its own strengths and limitations. DES is used to represent individual-level heterogeneity, primarily where there is an emphasis on the use of resources or queues [13]. DES was used to simulate the patient’s journey through the hospital, including isolation procedures, which consists of discrete events with probabilities causing competition for the agent’s next step (Additional file 1: Figure S1). A weakness of DES is the difficulty to include granular detail of the agents, which is a strength of ABS. ABS uses inductive and deductive reasoning to discover patterns of emergence [14]. ABS has three core concepts: agency, dynamics and structure [15]. In the OXA-181 hybrid model, agency gave the Patients, Beds and Wards individual identities. Dynamics allowed events occurring within the model to change when agents interacted with their environment. For example, a Patient’s colonization status becoming ‘detected colonized’ due to screening protocols. The structure of ABS is not programmed into the model, but emerges through the decisions of individual agents and their corresponding interactions. A hybrid model using ABS and DES was chosen to take advantage of both methodological strengths.

Many stochastic processes are involved in simulating a hospital outbreak. Each process in the model is driven by a random number generator (RNG) and its corresponding ‘seed’ that supplies the sequence of random numbers used in the course of each simulation. Each random choice made when executing the model will be the same across simulations when the ‘seed’ is held constant. Hospital processes have a major impact on a single simulation, and introduce major variability in outcomes. For example, a colonized patient being transferred to a long stay ward dramatically increases the potential for that patient to transmit the pathogen compared to being discharged. As the hospital processes causing the major variability are both known and moderately controllable, blocking, a design of experiment technique [16], was incorporated to introduce deterministic characteristics to the hybrid model. The deterministic characteristics were loosened and the model became more stochastic when the outbreak deviated from the calibrated path. This deviation occurs after the use or lack of use of WGS. Blocking is a technique which removes unnecessary variation through adding the nuisance variable as an independent variable in the model. The seeds for three controlled RNGs represent these nuisance variables: RNG one was used for the ward transfer probability and length of stay distributions. RNG two was used in the probability function which determines the number of new colonized patients at each time step. RNG three was used in choosing which patient was ‘randomly’ screened. These stochastic processes were kept consistent between scenarios, ensuring that outcomes are comparable. A fourth RNG which was not controlled was used in parameter variation in sensitivity analysis. It might seem counterintuitive for a hospital outbreak model to treat the spread and detection of the pathogen as nuisance factors, although the purpose of this model is to analyze the impact of pathogen WGS on the hospital management of the OXA-181 outbreak. In this case however, the conversion of the hospital processes to an easier to handle deterministic model [17] is a concession to the limited reliability and availability of information regarding hospital processes and the specific spread mechanisms of the pathogen. Even though the modelling of hospital processes and pathogen spread is stochastic, it does not capture the hospital’s operation closely enough to ensure the validity of the introduced resulting variability. Instead, this part of the model only serves as the machinery for building the backdrop of epidemic spread, against which to test the different screening regimes for this specific outbreak. Therefore, the aim of the calibration of this process is to reproduce the observed outbreak data closely, as opposed to representing the full range of possible outbreaks that can occur as a result of the simplified modelled processes.

The model allows all four agents to interact with each other and change state variables or characteristics based on those interactions (Fig. 1). The model’s major interactions were:

- ➢ Patient-Ward – when a Patient who is colonized enters a Ward agent it starts daily transmission calculations for the Ward agent.
- ➢ Patient-Bed – a Patient notifies the Bed of a detected colonization status so cleaning can occur after patient discharge.
- ➢ Patient-Floor – the Floor agent randomly screens Patients on a daily basis.
- ➢ Floor-Bed-Patient– the Floor can move a Patient from a Bed, when it is required for isolation.

### Initialization

Sourced from hospital admissions data, the model started with 551 existing hospital patients currently occupying beds. These patients were spread across 13 wards on four different floors (Table 1). The first colonized patient entered 1 day after initialization.

**Table 1:** Parameter description, values and sources used in the hybrid simulation model

Parameter	Value	Source
Initialization		
Initial starting population (n)	551	Hospital admissions dataset
Number of floors and wards	Level 5 (Ward A,B,C,D); Level 2 (Ward A,B,C,D,E); SIU; GARU (Bunya, Banksia, Cassia)	Building floor plans
Hospital Mechanics Sub Model		
Population entry rate, patients per day	24	Calibration
Ward admission, transfers and stays ^a^	see Additional file 1: Table S1-S3	Hospital admissions dataset
Susceptible-colonized sub model		
Transmission parameter *β*	Level 5 = 0.153, Level 2 = 0.14, SIU = 0.086, GARU = 0.086	Calibration
Outbreak Management Sub Model		
No WGS, outbreak number, patients with confirmed colonization	7–15	Expert opinion
No WGS, outbreak start delay, patients with confirmed colonization	2–5	Expert opinion
Microbiology test processing time, days	2	Expert opinion
Genome sequencing processing time, days (SD) ^b^	7 (0.5)	Expert opinion
Daily probability of patient being screened	Level 5 = 0.041, Level 2 = 0.043, GARU = 0.055, SIU = 0.056	Calibration
Genome sequencing cost, AU$ (SD) ^c^	354.70 (53.2)	Clinical records
Microbiology test cost, AU$(SD)	79.23 (11.88)	MBS item 69,306, PCR cost [35]
Bedroom cleaning cost, AU$(SD) ^d^	70 (10.5)	Clinical staff
Bed closure, AU$ (Q1-Q3)	216 (147–285)	Page et al., 2017 [21]
Hourly wage for infection control nurse, AU$ (SD)	40.33 (6.05)	Clinical staff & Queensland Health wage rates [36]
Executive infection control meeting ^e^, AU$	462.03 (69.3)	Clinical staff & Queensland Health wage rates [36]
Increased virulence scenario		
Infection chance	0.165	Tischendorf et al., 2016
Time till infection	27 days [11]	Tischendorf et al., 2016
Mortality (in-hospital)	0.40 (0.5)	Chang et al., 2015
Infection treatment costs, AU$	$2650	Chang et al., 2015
End-of-life costs, AU$	$19,696	Reeve et al., 2018
Environmental contamination scenario		
ET odds-ratio for patients in contaminated beds	2.65	Mitchell et al., 2015
Bed contamination length, days	5–10 days	Kramer et al., 2006

*GARU* geriatric assessment and recovery unit, *SIU* spinal injury unit, *SD* standard deviation, *PCR* polymerase chain reaction, ET environmental transmission

^a^Gamma distribution assigned

^b^Normal distribution assigned

^c^Comprising: sample prep 15.00, sequencing 105.00, analysis/storage 18.00, scientist 102.50, isolate handling 5.00, labor admin 33.33 biostats 75.85

^d^Hospital cleaning staff, labor hourly rate 31.24, curtains 33.00, consumables 5.00

^e^3 senior consultants 215.10, Infection control nurse 59.03, senior admin 65.10, manager 45.81

### Input data

The model does not use external input data to represent time-varying external processes.

### Sub-models

#### Hospital mechanics sub-model

The Hospital Mechanics sub-model was designed to simulate the patient journey throughout the hospital from day zero through to the end of the outbreak (Additional file 1: Figure S1). On day zero, an entry rate was used to continually re-supply patients to the wards. Patient movements were informed by the Queensland Health Admitted Patient Data Collection dataset. This provided data on admission ward, ward transfers and length of stay for all patients who were admitted to one of the 13 wards of interest between 1st April 2017 and 1st August 2017, a total of 4250 patient admissions. The ‘next ward’ parameter had a unique distribution for each ward pairing. This meant a patient’s journey through the hospital was always based on their previous ward. Ward stay was estimated as unique Gamma distributions for each ward pairing (current ward + next ward), using the ‘methods of moments’ approach [18]. A model ‘warm-up’ period was avoided because data was available on three patient movement parameters (initial ward, next ward, length of stay) from existing patients in the hospital at 1st April 2017 (day zero) and the patients entering the hospital after that date.

#### Outbreak management sub-model

The Outbreak Management sub-model governed the screening and isolation procedures in the model as informed by the state’s health department guidelines [19] and local expert opinion from the hospital’s microbiology and infection control nursing teams (Additional file 1: Figure S1). This sub-model has three phases:

- Pre-outbreak: Here patients were screened by culture-based and phenotypic microbiology tests. Patients in two specified wards were screened weekly and upon entry into the ward [19]. Random screening also occurred such that every Floor had a daily probability of the occupying patients getting screened. The random screening represented hospital staff searching for other pathogen outbreaks and patients that were routinely screened prior to specific procedures.
- Outbreak identification: An outbreak was typically declared when five patients were colonized, however, this threshold was lowered to one patient colonized if whole genome sequencing was routinely undertaken.
- Outbreak control: six processes were activated when an outbreak was confirmed:
  1. Patients were isolated in Level 5 Ward D, and once full, cohorting started in Level 5 Ward C. ‘Cohorting’ is where patients who are colonized room-in together. Patients in the geriatric and spinal injury units were isolated or cohorted on their own floors;
  2. All patients in the same room as a detected colonized Patient were screened;
  3. All beds with a colonized Patient were flagged for cleaning before another Patient entered the Bed;
  4. Executive meetings on outbreak control occurred daily, then weekly when fewer than five colonized Patients were detected per week;
  5. Patients in the geriatric and spinal injury units were screened weekly; and
  6. 29 days after the outbreak started, a hospital-wide screening took place replicating a real-life outbreak management action.

The Outbreak Management sub-model also accrued health system costs, which were calculated in 2018 Australian dollars (1 AUD = 0.69 USD [20]). Individual item costs were calculated through a detailed inventory of all resources used, and assigned to each microbiology test (US$55), WGS (US$246), bed closures (US$150) [21], bed cleaning (US$49), additional nursing time (US$28/h) and executive infection control meetings (US$320) (Table 1). While actual nursing hours may not increase during an outbreak, nursing staff are redirected to outbreak management duties such as contact precaution, patient isolation, environmental decontamination and wider patient screening activities. An opportunity cost of US$28 per hour was assigned to these duties. Executive meetings was costed at US$320 representing the opportunity cost of time spent by three senior medical consultants, an infection and prevention control nurse, senior administrator and a service manager.

#### Pathogen transmission sub-model

The Pathogen Transmission sub-model controlled the ward-level population transmission dynamics. Pathogen spread was based on the formula, $1-\exp \left\{\frac{-\beta C}{N}\right\}$ [22, 23] where β is the transmission parameter estimated by model calibration (Section 2.8), C is the number of colonized patients in the ward, N is the number of patients in the ward, excluding any patients who were isolated, and C and N values are updated daily. The transmission formula calculated the daily probability of a susceptible patient becoming colonized and is based on the frequency-dependent transmission term for stochastic epidemic models [22]. The number of patients colonized each day was calculated by as a binomial random variable, parameterized using the probability a single susceptible patient is colonized and the number of susceptible patients. The Patient parameter ‘Distance to closest colonized patient’ was used to select which patients were colonized, with smallest distance being selected.

### Model calibration

Empirical information on the OXA-181 transmission rate was unavailable. We therefore calibrated the transmission values required for the spread and detection of the pathogen to fit the model to reproduce the observed known colonizations in the real outbreak. The calibration involved 50,000 simulations of the OXA-181 hybrid model to find the smallest result of the calibration formula (Table 2). The calibration process measured the number of detected colonizations at day 69, 83 and 111 in each ward and the total across all four locations at the end of the outbreak. Days 69 and 83 were when there were spikes of colonization detection and day 111 was when the last detection occurred in the actual outbreak. The calibration formula aggregated the difference in colonization detections between the model and the real outbreak at each of the 13 floor-time point combinations, including total difference (Table 2). These dates were used to replicate the time it took to detect the pathogen within the hospital and then the speed of outbreak cessation once targeted infection control started. Note that hospital wide screening occurred in the real outbreak on day 85. The model was calibrated with a fixed WGS test turnaround time of 7 days and an outbreak starting number of five patients.

**Table 2:** Model calibration: Parameter variation range, calibration formulae and results

Parameter	Parameter calibration range^c^	Optimal calibration results	Calibration #2 results	Calibration #3 results
Level 2 Beta value^a^	0.001–0.2	0.14	0.094	0.139
Level 5 Beta value^a^	0.001–0.2	0.153	0.164	0.185
SIU Beta value^a^	0.001–0.2	0.086	0.068	0.08
GARU Beta value^a^	0.001–0.2	0.086	0.074	0.089
Lvl5 microbial test prob.^b^	0.01–0.07	0.041	0.055	0.04
Lvl2 microbial test prob.^b^	0.01–0.07	0.043	0.047	0.045
SIU microbial test prob.^b^	0.01–0.07	0.056	0.041	0.041
GARU microbial test prob.^b^	0.01–0.07	0.055	0.046	0.059
RNG 1 Seed	1–1000	701	457	952
RNG 2 Seed	1–1000	382	697	129
RNG 3 Seed	1–1000	465	810	348
Detected colonizations	Actual outbreak	Optimal calibration	Calibration #2	Calibration #3
Day 69: Lvl5, Lvl2, GARU, SIU	6, 1, 1, 1	7, 3, 0, 1	8, 0, 0, 1	10, 1, 1, 1
Day 83: Lvl5, Lvl2, GARU, SIU	11, 3, 1, 4	10, 6, 0, 1	16, 0, 1, 3	12, 1, 2, 1
Day 111: Lvl5, Lvl2, GARU, SIU	25, 13, 14, 8	21, 10, 5, 8	23, 0, 11, 6	19, 4, 12, 1
Total	72	75	75	73
Calibration Formulae^d^	abs (Lvl5 day69-AO Lvl5 day69) + abs (Lvl5 day83-AO Lvl5 day83) + abs (Lvl5 day111-AO Lvl5 day111) + abs (Lvl2 day69-AO Lvl2 day69) + abs (Lvl2 day83-AO Lvl2 day83) + abs (Lvl2 day111-AO Lvl2 day111) + abs (GARU day69-AO GARU day69) + abs (GARU day83-AO GARU day83) + abs(GARU day111-AO GARU day111) + abs(SIU day69-AO SIU day69) + abs(SIU day83-AO SIU day83) + abs(SIU day111-AO SIU day111) + abs(Total-AO Total)			

*Lvl5* Level five, *Lvl2* Level two, *GARU* Geriatric and Rehabilitation Unit, *SIU* Spinal injury unit, *AO* Actual outbreak, *RNG* random number generator, *abs* absolute value, *prob* Probability

^a^Beta value used in transmission formulae, $1-\exp \left\{\frac{-\beta C}{N}\right\}$

^b^The daily probability a patient in that floor will be randomly screened

^c^The values tested across 50,000 simulations

^d^Days 69 and 83 were chosen due spikes in colonization detections and day 111 was when the last detection occurred

### Simulation experiments

In total, seven scenarios were investigated as follows:

1. Actual Outbreak or WGS (late) – this was the base scenario of the actual OXA-181 outbreak in 2017 and the known cases of colonized patients. WGS was undertaken but only later in the outbreak as described above (Section 2.1.2).
2. No WGS – this scenario had no access to WGS and may be the common situation of most publicly government-funded hospitals in 2018. Without WGS information, a larger number of patients needs to be detected with the same pathogen strain and the infection control team needs additional time to review the information before declaring an outbreak.
3. WGS (early) – this was the optimal early sequencing scenario where WGS was used routinely with suspected patients, and an outbreak can be declared with the first positive anomalous (OXA-181) detection.
4. Environmental contamination and no WGS – this was the same as Scenario 2 but assumes that cleaning processes did not eradicate the pathogen. Hospital pathogens can persist on surfaces for months and can be a continuous source of transmission without regular preventive surface disinfecting [24]. In this scenario we allowed the Bed agent to become contaminated and contribute to the pathogen transmission. We assumed for each patient colonised there was a 50% chance of colonization spreading to other beds in same room and surviving on those beds for 5–10 days [24]. An environmental transmission odds ratio of 2.65 [25] was used to calculate the daily probability of a patient in a contaminated bed being colonized.
5. Environmental contamination and early WGS – this was the same as above but with early WGS.
6. Virulent and no WGS – this is Scenario 2 but where the pathogen is more harmful to patients by causing more invasive disease (e.g. bloodstream infection). The OXA-181 gene was carried on a highly mobile plasmid (a genetic element that can readily spread between bacterial strains and across species). This scenario represents the plasmid being present in pathogenic bacteria, with a higher proportion of colonized patients experiencing clinical disease, and standard therapy (e.g. beta-lactam antibiotics, including carbapenems) being compromised by the presence of the OXA-181 carbapenemase. In this scenario, we included a ‘colonization to infection’ probability of 0.17 [26] and a probability of death for infected patients of 0.40 [27]. The cost of US$1835 was used for treatment of infections [27] and US$13,640 for end-of-life care [28].
7. Virulent and early WGS – this is Scenario 3 plus the situation where the pathogen is more harmful to patients.

### Analyses

The outcomes included the: number of colonized patients; number of detected colonized patients; number of bed closures and accumulated hospital costs. Each scenario consisted of 1000 iterations. The model aggregated the sum of the events (using counters) and all outcomes that emerged from the interactions of the above sub-models and their probabilities, costs, assigned distributions and formula inputs. To address uncertainty of alternative calibration-derived values, one-way sensitivity analyses were performed using 1000 iterations for each model change. To address uncertainty of some inputs (e.g. cost of a bed closure or WGS) probabilistic sensitivity analyses were performed for each scenario.

## Results

### Calibration results

The parameters, the parameter variation, calibration formulae and calibration results are shown in Table 2. The second and third best calibration outcomes were presented to show the consistency of the calibration. The OXA-181 outbreak simulation successfully matched the real-life outbreak by starting with only 9–10 detections by day 69 and stopping the outbreak at 72 detected colonizations (Table 2).

### Evaluation of WGS strategies

Our analysis indicated that health system spending of US$19,469 on pathogen sequencing, compared with no WGS, avoided 77 OXA-181 colonizations and saved US$212,455 in outbreak resources (Table 3). The early WGS strategy (Scenario 3), compared with no WGS (Scenario 2), had US$485,836 in outbreak cost savings and 151 fewer colonizations. Early WGS was the dominant scenario in both the environmental contamination and virulent pathogen scenarios (Table 4). Early WGS predicted 40 fewer patients would become infected and US$559,710 of outbreak cost savings in the virulent pathogen scenario (Scenario 6 vs Scenario 7). The environmental contamination scenarios calculated 120 fewer detected colonizations and US$406,286 fewer costs (Scenario 4 vs Scenario 5). The largest cost component in each outbreak simulation was microbiology screening cost (varying from 44.3 to 89.2% of total costs) while the cost of sequencing was only 6% of the total costs in the actual outbreak (late WGS scenario, Scenario 2) and below 2% in scenarios with early WGS (Scenario 1).

**Table 3:** Result summaries for the outcomes measures from 1000 probabilistic simulations for three best calibrations

Total number of:	Optimal Calibration			Calibration #2			Calibration #3		
	Calibrated Real life^1^ (Scenario 1)	No WGS (Scenario 2)	Early WGS (Scenario 3)	Calibrated Real life^1^	No WGS	Early WGS	Calibrated Real life^1^	No WGS	Early WGS
Colonized patients (SD)	197	352 (170)	3 (0)	136	219 (90)	3 (0)	137	448 (320)	7 (9)
Detected patients (SD)	75	152 (75)	1 (0)	75	118 (48)	1 (0)	73	217 (136)	4 (7)
Sequencing tests (SD)	79		2 (0)	77		2 (0)	74		4 (7)
Bed closures (SD)	419	902 (486)	11 (2)	522	1145 (489)	5 (1)	428	1206 (767)	19 (28)
Total costs $US (SD)	318,654 (8406)	531,109 (234,315)	45,273 (3155)	349,160 (8889)	488,962 (178,224)	45,637 (2005)	322,981 (8441)	705,474 (360,758)	62,426 (38,507)
Whole genome sequencing costs	19,469 (393)		501 (51)	18,975 (388)		493 (10)	18,221 (385)		916 (1788)
Microbiology testing costs	180,933 (7804)	292,342 (110,451)	40,397 (2775)	193,338 (8169)	303,916 (104,391)	42,248 (1996)	186,432 (7862)	380,751 (160,304)	54,187 (28,663)
Cleaning costs	27,811 (1346)	58,075 (31,402)	733 (205)	33,125 (1488)	46,446 (20,638)	233 (43)	29,128 (1362)	78,749 (49,321)	1406 (1927)
Nursing costs	3091 (183)	6291 (3229)	44 (8)	3129 (184)	4818 (2068)	43 (2)	3000 (179)	8910 (5843)	157 (302)
Infection control executive meetings costs	24,366 (439)	38,873 (20,044)	1946 (124)	22,133 (401)	29,270 (12,413)	1925 (34)	21,803 (391)	55,779 (35,168)	2850 (2224)
Bed closure costs	62,984 (2481)	135,528 (72,750)	1652 (372)	78,460 (3012)	104,511 (47,699)	696 (104)	64,397 (2523)	181,286 (115,707)	2911 (4131)

NB: Empty cells denote where the outcome measures which were not modelled as part of the scenario

*SD* standard deviation, *US* United States, *WGS* whole genome sequencing

^1^No standard deviations were reported for non-cost outcome summaries in ‘Calibrated Real life’ as the model was calibrated using this scenario with a fixed outbreak signaling condition. Variation observed in ‘Calibrated Real life’ cost outcomes was due to the stochasticity of the cost parameters solely

**Table 4:** Result summaries for the outcomes measures from 1000 probabilistic simulations for environmental and virulent scenarios

Total number of:	Environmental contamination		Increased Virulence	
	No WGS (Scenario 4)	Early WGS (Scenario 5)	No WGS (Scenario 6)	Early WGS (Scenario 7)
Colonized patients (SD)	234 (179)	2 (0)	256 (157)	3 (0)
Env contamination sites (SD)	33 (28)	0 (0)		
Infected patients (SD)			41 (25)	1 (0)
Deaths (SD)			6 (5)	0 (0)
Detected patients (SD)	123 (86)	2 (0)	119 (70)	1 (0)
Sequencing tests (SD)		4 (0)		2 (0)
Bed closures (SD)	720 (508)	9 (1)	692 (424)	6 (2)
Total costs $US (SD)	451,280 (252,232)	44,994 (1954)	606,367 (318,967)	46,657 (2802)
Whole genome sequencing costs		985 (20)		502 (49)
Microbiology testing costs	259,910 (119,687)	40,141 (1950)	268,621 (114,042)	40,685 (2543)
Cleaning costs	46,310 (32,867)	476 (59)	44,328 (27,333)	370 (198)
Nursing costs	4982 (3698)	86 (5)	4843 (3034)	45 (9)
Infection control executive meetings costs	31,802 (22,710)	1925 (34)	31,369 (18,619)	1941 (101)
Bed closure costs	108,277 (76,740)	1381 (139)	104,019 (63,868)	915 (339)
Infection treatment costs			74,828 (46,413)	2200 (732)
Death costs			78,359 (61,973)	0 (0)

NB: Empty cells denote where the outcome measures which were not modelled as part of the scenario

*SD* standard deviation, *Env* Environmental, *US* United States, *WGS* whole genome sequencing

A sensitivity analysis based on the second and third best calibration results was undertaken to check the consistency of the main findings across different ‘seed’ settings. Each calibration represents an alternative OXA-181 outbreak pathway. This sensitivity analysis showed wide variation in the no WGS scenario, leading to variation in the base results: the early WGS scenario saved between US$440,000 to US$640,000 and between 117 to 213 in detected colonizations. However, the main conclusions of the study remains unchanged; early WGS was expected to reduce health system costs and avoid significant numbers of colonizations.

## Discussion

We combined two modeling methods, ABS and DES, to incorporate the process-driven features of a hospital environment with the individual details of patient-patient pathogen transmission. This is the first study to use a hybrid agent based/discrete event simulation model to investigate the impact of outbreak management interventions on the spread of pathogens throughout a hospital [6]. We created a flexible model capable of integrating a range of pathogen transmission features like environmental contamination, infection and mortality. We also were able to introduce a range of outbreak control policies such as hospital wide screening, cohorting and floor specific isolation protocols.

Using actual colonization and infection data to calibrate healthcare system models has been noted as a key aspect to improve future modeling [29]. A major strength of this study was the use of real-world colonisation data and patient ward transfer data over a 5 month outbreak to inform pathogen transmission and patient movement. We predicted that WGS was effective at diminishing the pathogen spread and lowering hospital outbreak costs. The key accumulated costs were microbiology culture tests and bed closure costs. Although WGS had the highest per item cost, it dramatically shortened the outbreak duration and limited the wider use of WGS.

Hybrid modeling provides clinicians with the information to assist decision-making in clinical situations and justify the use of new strategies. Healthcare system modeling is trending towards increased use of hybrid modeling, as the focus shifts from operational and process questions (patient flow, logistics and healthcare operations) to resources and design issues (capacity planning, resource allocation and program evaluation) [6]. A holistic healthcare simulation model must incorporate four interacting perspectives: resource allocation; health diffusion; population dynamics; and individual behavior [30]. The OXA-181 hybrid model encompassed these perspectives through features such as bed capacity, ward population dynamics and patient specific transmission. Future hybrid outbreak modeling could expand on the scope of work in our model by encompassing additional spillover effects. For example, controlling a hospital-infectious disease outbreak of a single pathogen has indirect impacts on the wider hospital system such as, increasing the number of isolation rooms available and avoiding delays in elective surgery.

Several limitations of this study are noted. Simulation modelling for hospital acquired infections are dominated by stochastic models [31] that are preferred when chance plays a large role of whether an event occurs, especially in smaller populations. We combined both stochastic and deterministic features in the model. A disadvantage of stochastic models in individual based simulation is the uncontrollable extent of randomness and the occurrence of stochastic fade-outs, where the outbreak fails to spread [32]. The deterministic characteristics of our model, introduced through calibrating the spread of the outbreak to real outbreak data, avoided stochastic fade-outs but possibly overestimated the number of colonizations. The OXA-181 model is not entirely a deterministic model, once the outbreak was identified the ongoing processes are stochastic as shown by the range of outcomes in Scenario 2. Expert opinion was required for a number of assumptions underpinning the scenarios we assessed, that is, no WGS, increased virulence and environmental contamination scenarios. Further validation using real-world data would be beneficial. The empirical findings of this model were context-specific and are unlikely to be generalizable to other bacterial outbreaks at other hospitals. However, the hybrid model can be adapted to other settings through its input driven ward and hospital level layout. For example, modeling across multiple bacterial outbreaks occurring concurrently is more realistic in a large metropolitan hospital, including daily use of sequencing, to support decisions to adopt WGS routinely as part of hospital policy. The data to inform such a simulation model was not available but future work on this is underway. Adapting this model to other outbreaks would require outbreak specific detection data to calibrate the model, ward transfer rates from hospital administration data and the corresponding hospital layout. In the hybrid model, WGS currently plays a role in identifying strains and identifying the source of outbreaks. Antimicrobial susceptibility was not parameterized in the hybrid model because the clinical utility and application of WGS is still emerging [33]. Future work is required to model in what capacity WGS can change infection control and prevention.

Several lessons were learned regarding the construction and calibration of the complex and detailed OXA-181 hybrid model. Recreating a real-life outbreak to the detail of patient ward movement created a model vulnerable to stochastic variability and ‘noise’. Blocking was successfully used to limit the outbreak variability [16]. Although less computationally intense approaches in reducing the variability in patient movement across simulations is worth exploring. Each hospital outbreak simulation model has to take into account the different possible transmission pathways, outbreak control measures and patient risk factors specific to the causative pathogen. Depending on the pathogen there are up to five transmission routes, seven outbreak control measures, four hospital processes and four infection spreading possesses that could be included in a hospital outbreak model [34]. The elements to include need to be thoroughly considered to ensure a parsimonious model but still captures all essential outbreak components necessary for reporting.

In conclusion, we incorporated agent-based, discrete-event and geospatial system modeling to develop a hybrid simulation model for evaluating a hospital outbreak. We used the model to quantify the economic and clinical impact of introducing whole genome sequencing into HAI outbreak management. Early WGS was predicted to contain an *E. coli* OXA-181 outbreak and reduced hospital costs. This study highlights the strengths and limitations of using hybrid modeling to evaluate hospital outbreak interventions.

## Supplementary information

**Additional file 1: Figure S1.** Hospital mechanics and outbreak management sub-models. **Table S1.** Hospital entry (first ward) distribution of existing patients and new admission. **Table S2.** Number, proportion and ward length of stay estimates of the different ward pair combinations for existing patients. **Table S3.** Number, proportion and ward length of stay estimates of the different ward pair combinations for new admissions.

## Data Availability

The data that support the findings of this study are available from Queensland Health but restrictions apply to the availability of these data, which were used under license for the current study, and so are not publicly available. All variables generated from this data are included this article and its supplementary files.

## Abbreviations

ABS: agent-based simulation
DES: discrete-event simulation
HAI: healthcare-associated infections
RNG: random number generator
WGS: whole genome sequencing
