# Supplementary material for: A hybrid simulation model approach to examine bacterial genome sequencing during a hospital outbreak
Source: BMC Infect Dis. 2020 Jan 23;20:72. doi: 10.1186/s12879-019-4743-3 (PMC6979342; doi:10.1186/s12879-019-4743-3)
Supplement: Supplementary file 1 — Additional file 1: Figure S1. Hospital mechanics and outbreak management sub-models. Table S1. Hospital entry (first ward) distribution of existing patients and new admission. Table S2. Number, proportion and ward length of stay estimates of the different ward pair combinations for existing patients. Table S3. Number, proportion and ward length of stay estimates of the different ward pair combinations for new admissions. [file 12879_2019_4743_MOESM1_ESM.docx]

Supplementary Materials for manuscript ‘A hybrid simulation model approach to examine bacterial genome sequencing during a hospital outbreak.’

Authors: Thomas M Elliott, Xing J. Lee, Anna Foeglein, Patrick N. Harris, Louisa G Gordon

**Calibration details:**

The calibration targets are represented within the calibration formulae, the simulation which matches the targets most closely was chosen. The calibration formulae calculated the difference between the number of colonisation detections in the ‘real outbreak’ and the simulation at 13 specific floor-time points. These time points were day 69, 83 and 111. Days 69 and 83 were used due to spikes in colonization detection and around the hospital wide screening. Day 111 was when the last detection occurred in the actual outbreak. These dates were used to replicate the time it took to detect the pathogen within the hospital and then the speed of outbreak cessation once targeted infection control started. There were 11 parameters varied in the calibration; the beta values for each floor, the probability that a patient will be randomly screened in each floor and the three random number generator seeds.

Figure S1: Hospital mechanics and outbreak management sub-models

| 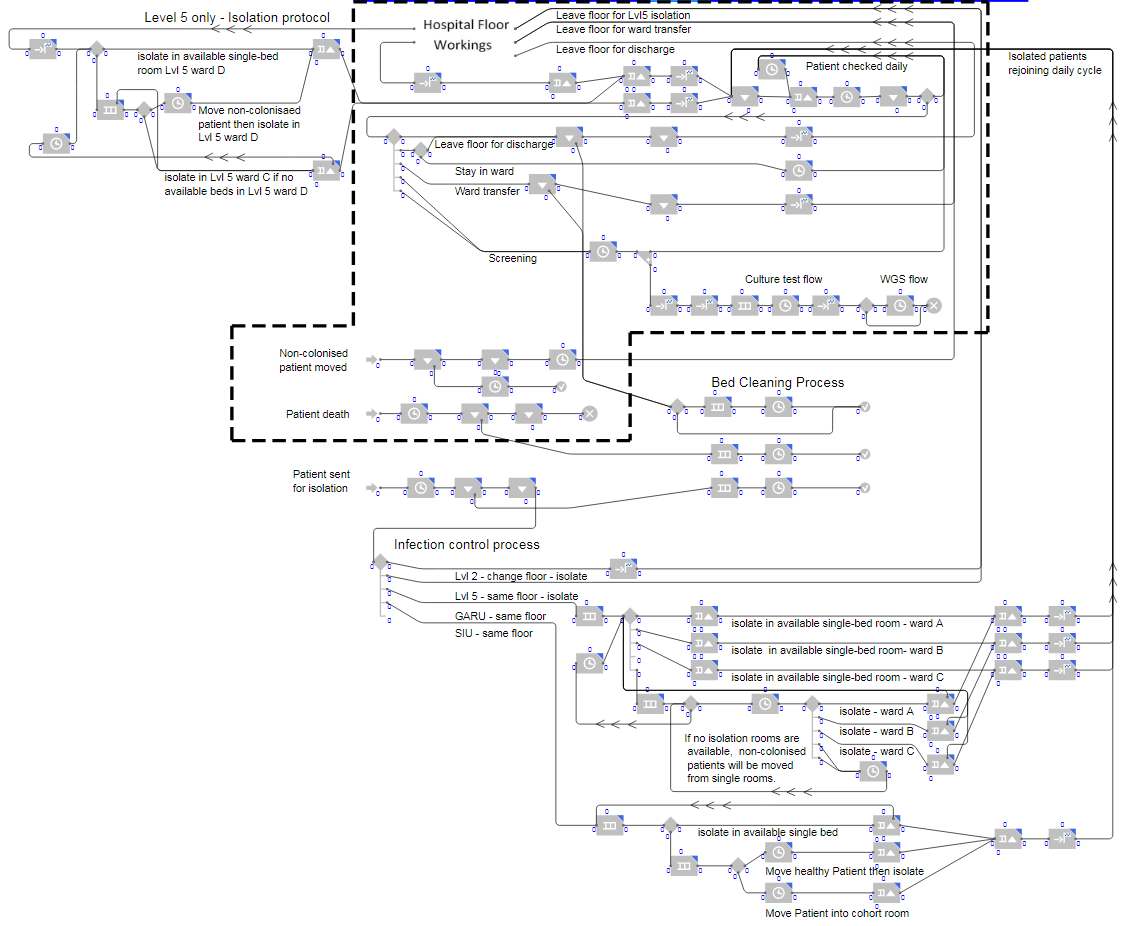 | Event Key | Description |
| --- | --- | --- |
|  | Queue: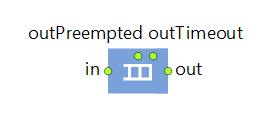 | Patients will wait until the next event is ready to take them |
|  | Delay: 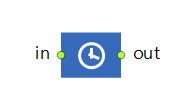 | Patients will wait for a specified period of time. |
|  | Select output: 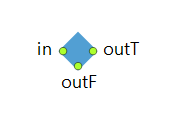 | Patients are set on different paths pending if a condition is true |
|  | Select output5: 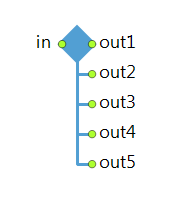 | Patients are set on different paths pending if a mutually exclusive condition is true |
|  | Move To: 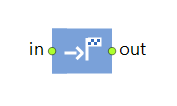 | Moves patients on the GIS map |
|  | Seize: 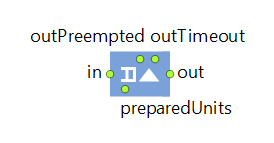 | Seize agents such as Beds, nurse, physicians |
|  | Release: 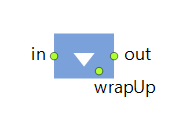 | Releases agents. Wrap up function allows actions to occur to the agent before it can be seized again. |
|  | Enter: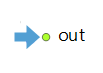 | Patients are programmatically told to enter this pathway. |

Note: The events inside the dashed black box make up the hospital mechanics sub-model and the events outside is the outbreak management sub-model

Table S1: Hospital entry (first ward) distribution of existing patients and new admission.

|  | Percentage of admissions (%) | |
| --- | --- | --- |
| Ward | Existing patients | New admissions |
| BANK | 3.9 | 1.6 |
| BUNY | 4.7 | 1.2 |
| CASS | 4.5 | 2 |
| other | 44.2 | 18.8 |
| SIU | 6.6 | 0.9 |
| W2A | 4.1 | 9.6 |
| W2B | 3.4 | 8.3 |
| W2C | 5.9 | 11.3 |
| W2D | 4.5 | 10 |
| W2E | 4.5 | 11.5 |
| W5A | 3.8 | 6.8 |
| W5B | 3.2 | 6.7 |
| W5C | 3.4 | 6.8 |
| W5D | 3.4 | 4.7 |

Notes:

- Ward ‘other’ covers all other hospital wards excluding the 13 study wards.
- Same day patients were not included due to the low chance of transmission.
- Patients whose ‘Initial ward’ was emergency room had their ward corrected to the second ward they were admitted into, due to patients not staying in emergency room for more than 1 day.

Table S2: Number, proportion and ward length of stay estimates of the different ward pair combinations for existing patients

| Ward transfer pairing | | Number of transfers observed | Transfer proportion | ward stay, days (SD) | Gamma distribution estimates | |
| --- | --- | --- | --- | --- | --- | --- |
| from | to |  |  |  | shape | scale |
| BANK | discharged | 32 | 0.89 | 31.3 (28) | 1.2 | 25.1 |
| BANK | other | 4 | 0.11 | 19.3 (16.7) | 1.3 | 14.6 |
| BUNY | discharged | 35 | 0.9 | 36.4 (28.9) | 1.6 | 22.9 |
| BUNY | other | 4 | 0.1 | 12.3 (11.1) | 1.2 | 10 |
| CASS | discharged | 29 | 1 | 31.4 (22.6) | 1.9 | 16.3 |
| other | BANK | 5 | 0.02 | 15.6 (12.5) | 1.6 | 10 |
| other | BUNY | 11 | 0.03 | 20.4 (16.5) | 1.5 | 13.4 |
| other | discharged | 286 | 0.87 | 13.8 (26.6) | 0.3 | 51.1 |
| other | W2A | 5 | 0.02 | 5.6 (3.4) | 2.7 | 2.1 |
| other | W2C | 4 | 0.01 | 17.8 (19.3) | 0.8 | 21 |
| other | W2D | 6 | 0.02 | 12.3 (8.8) | 2 | 6.3 |
| other | W5D | 12 | 0.04 | 9.5 (9.3) | 1.1 | 9 |
| SIU | discharged | 39 | 1 | 87.5 (83.5) | 1.1 | 79.6 |
| W2A | BANK | 3 | 0.13 | 18.7 (18.2) | 1.1 | 17.7 |
| W2A | discharged | 9 | 0.38 | 10 (12.5) | 0.6 | 15.5 |
| W2A | other | 12 | 0.5 | 5.8 (4.3) | 1.8 | 3.2 |
| W2B | discharged | 13 | 0.68 | 13.7 (14.5) | 0.9 | 15.4 |
| W2B | other | 6 | 0.32 | 18.2 (17.5) | 1.1 | 16.9 |
| W2C | discharged | 14 | 0.4 | 20.3 (42.4) | 0.2 | 88.8 |
| W2C | other | 21 | 0.6 | 21.4 (37.5) | 0.3 | 65.5 |
| W2D | discharged | 26 | 0.9 | 7.1 (11.7) | 0.4 | 19.3 |
| W2D | other | 3 | 0.1 | 4 (1.7) | 5.3 | 0.8 |
| W2E | discharged | 22 | 0.88 | 9.5 (8.7) | 1.2 | 7.9 |
| W2E | other | 3 | 0.12 | 7 (5.3) | 1.8 | 4 |
| W5A | discharged | 16 | 0.59 | 13.4 (12.7) | 1.1 | 12.1 |
| W5A | other | 7 | 0.26 | 11.4 (10.7) | 1.1 | 10 |
| W5A | W5B | 4 | 0.15 | 46 (75) | 0.4 | 122.2 |
| W5B | discharged | 12 | 0.6 | 38.8 (55.1) | 0.5 | 78.3 |
| W5B | other | 5 | 0.25 | 21.2 (23) | 0.8 | 25 |
| W5B | W5A | 3 | 0.15 | 18 (13.7) | 1.7 | 10.5 |
| W5C | discharged | 12 | 0.71 | 16.3 (15.5) | 1.1 | 14.7 |
| W5C | other | 5 | 0.29 | 13.8 (9.9) | 1.9 | 7.1 |
| W5D | discharged | 14 | 0.47 | 16 (14.5) | 1.2 | 13.1 |
| W5D | other | 16 | 0.53 | 12.6 (11.4) | 1.2 | 10.3 |

Table S3: Number, proportion and ward length of stay estimates of the different ward pair combinations for new admissions

| Ward transfer pairing | | Number of transfers observed | Transfer proportion | ward stay, days (SD) | Gamma distribution estimates | |
| --- | --- | --- | --- | --- | --- | --- |
| from | to |  |  |  | shape estimate | scale estimate |
| BANK | discharged | 126 | 0.82 | 22.6 (25.8) | 0.8 | 29.5 |
| BANK | other | 24 | 0.16 | 3.7 (3.9) | 0.9 | 4.2 |
| BANK | W2D | 3 | 0.02 | 2.3 (0.6) | 16.3 | 0.1 |
| BUNY | discharged | 101 | 0.83 | 25.8 (29.1) | 0.8 | 32.9 |
| BUNY | other | 17 | 0.14 | 7.9 (15.3) | 0.3 | 29.9 |
| BUNY | W2C | 4 | 0.03 | 17.5 (29) | 0.4 | 48.2 |
| CASS | discharged | 138 | 0.77 | 24.4 (34.5) | 0.5 | 48.7 |
| CASS | other | 38 | 0.21 | 3.4 (4.3) | 0.6 | 5.6 |
| CASS | W2B | 4 | 0.02 | 2.8 (1) | 8.3 | 0.3 |
| other | BANK | 21 | 0.01 | 24.1 (35.9) | 0.5 | 53.4 |
| other | BUNY | 28 | 0.01 | 13.9 (11.5) | 1.5 | 9.6 |
| other | CASS | 26 | 0.01 | 12.4 (11.6) | 1.1 | 10.9 |
| other | discharged | 1105 | 0.54 | 4.6 (10) | 0.2 | 21.8 |
| other | SIU | 4 | 0 | 15 (19.1) | 0.6 | 24.2 |
| other | W2A | 67 | 0.03 | 4.5 (4.9) | 0.9 | 5.3 |
| other | W2B | 50 | 0.02 | 6.2 (7) | 0.8 | 8 |
| other | W2C | 115 | 0.06 | 6.7 (6.2) | 1.2 | 5.8 |
| other | W2D | 226 | 0.11 | 5.2 (6.2) | 0.7 | 7.4 |
| other | W2E | 53 | 0.03 | 5.7 (4.3) | 1.7 | 3.3 |
| other | W5A | 112 | 0.05 | 3.9 (4.8) | 0.6 | 6 |
| other | W5B | 78 | 0.04 | 3.5 (2.7) | 1.7 | 2.1 |
| other | W5C | 91 | 0.04 | 3.7 (2.2) | 2.7 | 1.4 |
| other | W5D | 67 | 0.03 | 8.7 (10.8) | 0.7 | 13.3 |
| SIU | discharged | 55 | 0.85 | 71.5 (72.8) | 1 | 74.1 |
| SIU | other | 10 | 0.15 | 13.7 (35.9) | 0.1 | 94.3 |
| W2A | BANK | 13 | 0.03 | 8.8 (4.8) | 3.3 | 2.6 |
| W2A | BUNY | 9 | 0.02 | 14.3 (8.8) | 2.7 | 5.4 |
| W2A | CASS | 11 | 0.02 | 7.6 (2.9) | 7.1 | 1.1 |
| W2A | discharged | 309 | 0.62 | 6.2 (7.2) | 0.7 | 8.4 |
| W2A | other | 132 | 0.27 | 8.3 (9.2) | 0.8 | 10.1 |
| W2A | SIU | 10 | 0.02 | 16.1 (8.1) | 3.9 | 4.1 |
| W2A | W2B | 7 | 0.01 | 3.7 (1.4) | 7.2 | 0.5 |
| W2A | W2C | 5 | 0.01 | 5.8 (6.8) | 0.7 | 8.1 |
| W2B | BANK | 21 | 0.05 | 8.5 (4.5) | 3.6 | 2.4 |
| W2B | BUNY | 11 | 0.03 | 10.1 (6.9) | 2.1 | 4.8 |
| W2B | CASS | 8 | 0.02 | 8.1 (5.4) | 2.2 | 3.6 |
| W2B | discharged | 278 | 0.65 | 5.5 (7) | 0.6 | 8.9 |
| W2B | other | 98 | 0.23 | 9 (8.6) | 1.1 | 8.1 |
| W2B | SIU | 10 | 0.02 | 14.9 (6.3) | 5.5 | 2.7 |
| W2B | W2C | 3 | 0.01 | 5.3 (5.8) | 0.9 | 6.3 |
| W2C | BANK | 9 | 0.01 | 14.1 (9.5) | 2.2 | 6.4 |
| W2C | BUNY | 6 | 0.01 | 8.7 (6.6) | 1.7 | 5 |
| W2C | CASS | 26 | 0.04 | 7 (4.2) | 2.8 | 2.5 |
| W2C | discharged | 302 | 0.48 | 6 (7.1) | 0.7 | 8.5 |
| W2C | other | 230 | 0.37 | 8 (6.7) | 1.4 | 5.6 |
| W2C | W2A | 14 | 0.02 | 6.8 (6.2) | 1.2 | 5.6 |
| W2C | W2B | 15 | 0.02 | 6.9 (5.3) | 1.7 | 4 |
| W2C | W2E | 7 | 0.01 | 6.1 (3.3) | 3.5 | 1.8 |
| W2C | W5A | 4 | 0.01 | 6 (2.2) | 7.7 | 0.8 |
| W2C | W5C | 10 | 0.02 | 9.9 (6.4) | 2.4 | 4.1 |
| W2D | BANK | 3 | 0 | 4.3 (3.2) | 1.8 | 2.4 |
| W2D | BUNY | 5 | 0.01 | 6 (3.2) | 3.4 | 1.8 |
| W2D | CASS | 4 | 0.01 | 8.3 (7.5) | 1.2 | 6.8 |
| W2D | discharged | 472 | 0.67 | 5.3 (6.7) | 0.6 | 8.5 |
| W2D | other | 175 | 0.25 | 5.1 (3.5) | 2.2 | 2.4 |
| W2D | SIU | 5 | 0.01 | 3.8 (1.3) | 8.5 | 0.4 |
| W2D | W2C | 3 | 0 | 7.3 (4.9) | 2.2 | 3.3 |
| W2D | W2E | 20 | 0.03 | 4.2 (2.3) | 3.4 | 1.2 |
| W2D | W5B | 3 | 0 | 10.3 (4.2) | 6.2 | 1.7 |
| W2D | W5D | 11 | 0.02 | 9.5 (8.9) | 1.2 | 8.2 |
| W2E | discharged | 460 | 0.8 | 6.2 (6.5) | 0.9 | 6.7 |
| W2E | other | 88 | 0.15 | 7.7 (7.3) | 1.1 | 6.9 |
| W2E | W2C | 4 | 0.01 | 3.5 (0.6) | 36.8 | 0.1 |
| W2E | W2D | 14 | 0.02 | 4.1 (2.1) | 3.9 | 1 |
| W2E | W5D | 8 | 0.01 | 4.3 (2.7) | 2.5 | 1.7 |
| W5A | BANK | 6 | 0.01 | 12.5 (7.2) | 3 | 4.2 |
| W5A | BUNY | 4 | 0.01 | 7.5 (5) | 2.3 | 3.3 |
| W5A | CASS | 7 | 0.02 | 9.6 (10.7) | 0.8 | 12 |
| W5A | discharged | 277 | 0.61 | 7.2 (11.3) | 0.4 | 17.9 |
| W5A | other | 104 | 0.23 | 7.7 (7.8) | 1 | 7.9 |
| W5A | W2A | 4 | 0.01 | 12.5 (17) | 0.5 | 23.2 |
| W5A | W2D | 5 | 0.01 | 4.4 (4.3) | 1.1 | 4.2 |
| W5A | W2E | 3 | 0.01 | 3 (1) | 9 | 0.3 |
| W5A | W5B | 15 | 0.03 | 16.5 (19.2) | 0.7 | 22.4 |
| W5A | W5C | 22 | 0.05 | 12.1 (17.8) | 0.5 | 26.2 |
| W5A | W5D | 5 | 0.01 | 17.2 (16.6) | 1.1 | 16 |
| W5B | BANK | 8 | 0.02 | 10.5 (9.3) | 1.3 | 8.2 |
| W5B | CASS | 5 | 0.01 | 7.4 (2.7) | 7.5 | 1 |
| W5B | discharged | 237 | 0.6 | 5.2 (4.7) | 1.2 | 4.2 |
| W5B | other | 103 | 0.26 | 8.1 (8.4) | 0.9 | 8.6 |
| W5B | W2D | 3 | 0.01 | 3 (1) | 9 | 0.3 |
| W5B | W5A | 23 | 0.06 | 11.3 (17) | 0.4 | 25.5 |
| W5B | W5C | 19 | 0.05 | 5.2 (4.7) | 1.2 | 4.3 |
| W5C | BANK | 6 | 0.01 | 7.8 (5.8) | 1.8 | 4.3 |
| W5C | BUNY | 6 | 0.01 | 17.3 (7.6) | 5.2 | 3.3 |
| W5C | CASS | 9 | 0.02 | 9 (5) | 3.2 | 2.8 |
| W5C | discharged | 253 | 0.58 | 5.9 (8.4) | 0.5 | 11.9 |
| W5C | other | 108 | 0.25 | 9.9 (16.4) | 0.4 | 27.2 |
| W5C | W2D | 5 | 0.01 | 4.4 (4.3) | 1 | 4.3 |
| W5C | W5A | 22 | 0.05 | 5.2 (4.4) | 1.4 | 3.6 |
| W5C | W5B | 18 | 0.04 | 13.9 (22.8) | 0.4 | 37.4 |
| W5C | W5D | 6 | 0.01 | 5 (3.2) | 2.4 | 2.1 |
| W5D | BANK | 3 | 0.01 | 23.3 (31) | 0.6 | 41.2 |
| W5D | BUNY | 3 | 0.01 | 21.3 (26.6) | 0.6 | 33.3 |
| W5D | CASS | 3 | 0.01 | 17 (14.2) | 1.4 | 11.8 |
| W5D | discharged | 137 | 0.46 | 5.5 (6) | 0.8 | 6.6 |
| W5D | other | 115 | 0.38 | 7.9 (8.2) | 0.9 | 8.6 |
| W5D | W2A | 3 | 0.01 | 4 (2) | 4 | 1 |
| W5D | W2B | 3 | 0.01 | 5.7 (1.5) | 13.8 | 0.4 |
| W5D | W2C | 4 | 0.01 | 5 (1.8) | 7.5 | 0.7 |
| W5D | W2D | 17 | 0.06 | 7.8 (9.3) | 0.7 | 11.1 |
| W5D | W2E | 4 | 0.01 | 4.8 (2.6) | 3.3 | 1.5 |
| W5D | W5A | 3 | 0.01 | 10.3 (5) | 4.2 | 2.5 |
| W5D | W5C | 4 | 0.01 | 6.3 (4.4) | 2 | 3.1 |
